# Off‐axis response due to mechanical coupling across all six degrees of freedom in the human disc

**DOI:** 10.1002/jsp2.1047

**Published:** 2019-03-22

**Authors:** John F. DeLucca, Dhara Amin, John M. Peloquin, Edward J. Vresilovic, John J. Costi, Dawn M. Elliott

**Affiliations:** ^1^ Department of Biomedical Engineering University of Delaware Newark Delaware; ^2^ Biomechanics and Implants Research Group, The Medical Device Research Institute College of Science and Engineering, Flinders University Adelaide Australia; ^3^ UPMC Pinnacle Lititz Pennsylvania

**Keywords:** coupled motion, hybrid control, intervertebral disc, off‐axis, spine loading

## Abstract

The kinematics of the intervertebral disc are defined by six degrees of freedom (DOF): three translations (Tz: axial compression, Tx: lateral shear, and Ty: anterior‐posterior shear) and three rotations (Rz: torsion, Rx: flexion‐extension, and Ry: lateral bending). There is some evidence that the six DOFs are mechanically coupled, such that loading in one DOF affects the mechanics of the other five “off‐axis” DOFs, however, most studies have not controlled and/or measured all six DOFs simultaneously. Additionally, the relationships between disc geometry and disc mechanics are important for evaluation of data from different sized donor and patient discs. The objectives of this study were to quantify the mechanical behavior of the intervertebral disc in all six degrees of freedom (DOFs), measure the coupling between the applied motion in each DOF with the resulting off‐axis motions, and test the hypothesis that disc geometry influences these mechanical behaviors. All off‐axis displacements and rotations were significantly correlated with the applied DOF and were of similar magnitude as physiologically relevant motion, confirming that off‐axis coupling is an important mechanical response. Interestingly, there were pairs of DOFs that were especially strongly coupled: lateral shear (Tx) and lateral bending (Ry), anterior‐posterior shear (Ty) and flexion‐extension (Rx), and compression (Tz) and torsion (Rz). Large off‐axis shears may contribute to injury risk in bending and flexion. In addition, the disc responded to shear (Tx, Ty) and rotational loading (Rx, Ry, and Rz) by increasing in disc height in order to maintain the applied compressive load. Quantifying these mechanical behaviors across all six DOF are critical for designing and testing disc therapies, such as implants and tissue engineered constructs, and also for validating finite element models.

## 1 INTRODUCTION

**Figure 1:**
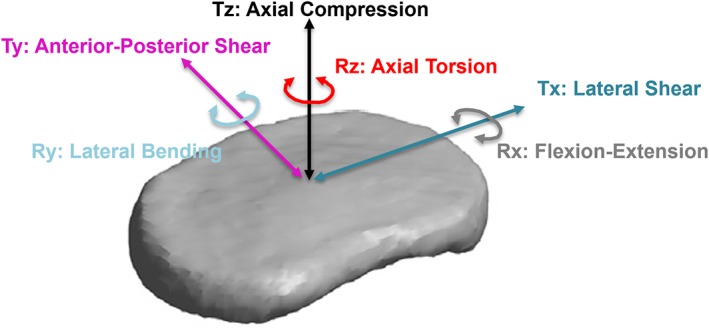
Schematic of axes of loading for the intervertebral disc and labels for each degree‐of‐freedom. T = translation and R = rotation. (Figure modified from Peloquin et al^1^.)

The kinematics of the intervertebral disc are defined by six degrees of freedom (DOF): three translations (T) (Tz: axial compression, Tx: lateral shear, and Ty: anterior‐posterior shear) and three rotations (R) (Rz: torsion, Rx: flexion‐extension, and Ry: lateral bending), (Figure 1, adapted from^1^). Mechanical tests using multiaxial testing systems such as pulleys and weights,^2^ cables and linear actuators,^3^, ^4^ stepper motors and linear bearings,^5^, ^6^ and Stewart platforms (hexapods)^7^, ^8^, ^9^ have demonstrated that the disc's load‐deformation and moment‐rotation responses are highly nonlinear and viscoelastic. Quantifying these mechanical behaviors across all six DOF are critical for designing and testing disc therapies, such as implants and tissue engineered constructs^10^, ^11^ and also for validating finite element models.^8^ Moreover, disc multi‐axial mechanics drive many critical physiological processes under investigation, such as low back pain; tears and herniation; injury, repair and remodeling; and cell mechanotransduction.^12^, ^13^, ^14^

The disc mechanical response has been measured in all DOFs; however, most studies apply either position control or pure moment control in only one or two DOFs, such as axial compression^15^, ^16^, ^17^ or axial torsion.^18^, ^19^, ^20^ Most studies do not control or measure all six DOFs simultaneously, meaning that the off‐axis DOF are ignored. Importantly, there is evidence that the six DOFs are mechanically coupled, such that loading in one DOF affects the mechanics of the other five “off‐axis” DOFs.^21^ For example, applying axial compression increases stiffness in the rotational DOFs (Rx, Ry, Rz).^22^, ^23^ In this study, we sequentially tested the disc's response to cyclic loading in each of the six DOFs, using a state‐of‐the‐art hexapod system to measure both same‐axis and off‐axis responses.^9^, ^24^, ^25^ We hypothesized that applied loading in each DOF would generate coupled off‐axis motions (translations and rotations) in the other five DOFs, and we tested this hypothesis by correlating, for each DOF, the applied motion vs measurements of the resulting five off‐axis motions.

Relationships between disc geometry and disc mechanics, including coupled off‐axis responses, are important for evaluation of data from different sized donor and patient discs, interpretation of finite element models, and development of patient‐specific models. Geometry, such as height and width, is known to affect the same‐axis mechanical response of a disc segment when loading is applied to a DOF.^26^, ^27^ For example, in a computational parametric simulation, disc height correlated with range of motion in pure moment flexion‐extension (Rx), lateral bending (Ry), and axial torsion (Rz).^27^ In this study, we hypothesized that coupled off‐axis motions for each DOF are also, like the same‐axis mechanical response, correlated with disc geometry.

Therefore, the objectives of this study were to quantify the mechanical behavior of the intervertebral disc in all six degrees of freedom (DOFs), measure the coupling between each applied motion in each DOF and the resulting off‐axis motions, and test the hypothesis that disc geometry influences these mechanical behaviors.

## 2 METHODS

### 2.1 Specimens

Human lumbar intervertebral disc bone‐disc‐bone segments (*n* = 8) from five spines (three male and two female; age 49 ± 9 years, range 35‐59) were dissected with posterior elements removed and graded for degeneration (Levels: L1‐L2 (*n* = 1), L2‐L3 (*n* = 1), L3‐L4 (*n* = 2), L4‐L5 (*n* = 4)). To limit variation from degeneration, only mild to moderately degenerated discs (Pfirrmann grades 2 to 3) were used. The disc's anterior‐posterior width (W_AP_), lateral width (W_LAT_), and disc height were determined from lateral and coronal X‐ray images. The disc's cross‐sectional area was calculated from A = 0.84 * W_AP_ * W_LAT._ 28 The disc's aspect ratio was calculated as W_LAT_/W_AP_. Prior to testing, discs were thawed overnight under 50 N axial compression in 0.15 M PBS with a cocktail of protease inhibitors, antibacterial, and antifungal agents: Amphotericin B—2 mL (10 mL/L), Benzamidine—32 mg (1 mM), EDTA—4 mL (1 mM), Idoacetamide—4 mL (1 mM), Gentamicin—10 mg (10 μg/mL), and Pepstatin A—3 mL (0.3 μM).^24^ Three k‐wires were inserted halfway into the sides of each vertebra, 120° apart, and the vertebrae were potted into custom cups using polymethylmethacrylate (bone cement). During potting, the lordotic angle measured from the x‐ray images was maintained using an alignment device and plastic wedges as previously described.^9^ The potted bone‐disc‐bone segment specimen was then mounted in a 6DOF hexapod robot with a 37°C 0.15 M PBS bath for mechanical testing.

### 2.2 Mechanical testing

**Figure 2:**
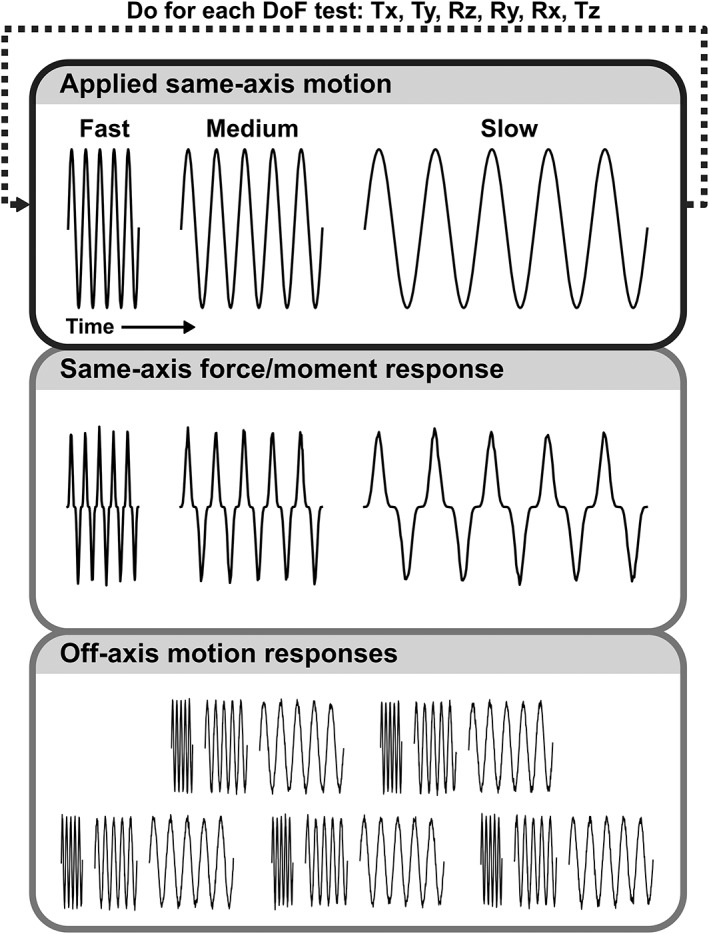
Schematic for mechanical testing protocol. Testing consisted of an overnight 0.2 MPa compression preload followed by Degree of Freedom (DOF) tests in which 5 cycles each at 0.5, 0.05, and 0.005 Hz (fast, medium, and slow rates) were performed for each DOF, followed by a 30 minute recovery period. To maintain off‐axis zero force and moment conditions, the off‐axes displace and rotate in response to loading. DOF test order was Tx (lateral shear), Ty (anterior–posterior shear), Rz (axial torsion), Ry (lateral bending), and Rx (flexion‐extension). Finally, the Tz (axial compression) test was performed with 5 cycles at 1, 0.1, and 0.01 Hz with recovery after 0.1 Hz as well as after the 0.01 Hz test

Segments were tested using a state‐of‐the‐art hexapod system that simultaneously controls and measures all six DOFs.^9^, ^24^, ^25^ The protocol consisted of an axial compression preload followed by six sequential tests, subsequently called “DOF tests,” each of which applied cyclic loading to one target DOF (Figure 2). A novel hybrid control system was used, for details of the apparatus and control system, please see previous publications.^9^, ^24^, ^25^, ^29^ In each DOF test, prescribed cyclic loading was applied to the target DOF in position or load control, and the off‐axis DOFs were load controlled in real time to maintain (approximately) zero off‐axis forces and moments.^29^ This adaptive approach to minimizing off‐axis forces and moments allows the disc's center of rotation to change during the protocol, which is an advantage over displacement controlled tests that fix the center of rotation to one location, potentially causing supra‐physiologic and damaging loads.^21^, ^29^

The preload consisted of axial compression with off‐axis forces and moments controlled to zero. It was held overnight for 12 hours before the DOF tests started. The preload was chosen to produce a nucleus pulposus (NP) pressure of 0.2 MPa, which mimicked physiologic conditions and reduced axial displacement creep during the subsequent DOF tests. The preload force was calculated as $F=\frac{P*A}{1.5}$, where *P* is the desired NP pressure (0.2 MPa), *A* is the disc cross‐sectional area, and 1.5 is an empirically derived factor.^24^ The axial preload was maintained during through the DOF tests except (as described below) when axial force was cyclically varied in the Tz test.

Six “DOF tests”, each of which applied cyclic loading to a specific DOF and controlled the off‐axis moments and forces to be zero (hybrid control), were performed in the sequence Tx, Ty, Rz, Ry, Rx, and Tz (Figure 2). As testing sequence can influence disc mechanics, the specific order used was chosen to minimize disc volume lost throughout testing.^25^ Each DOF test was separated from the others by a 30 minute recovery period (held in the preload state). Each DOF test consisted of a three blocks of five loading cycles at fast, medium, then slow cycle frequencies. In the Tx, Ty, Rz, Ry, and Rx tests, the blocks' frequencies were 0.5, 0.05, and 0.005 Hz, and the target DOF was sinusodially loaded in displacement or rotation control. In the Tz test, the blocks' frequencies were 1, 0.1, and 0.01 Hz, and Tz was loaded with haversine cycles in force control. Loading in Tz caused significant axial creep, which is why the Tz test was done in force control. An additional 30 minute recovery period was also inserted after the Tz medium rate block to allow some recovery of axial creep before the slow rate block started. The Tx and Ty tests had a slightly modified hybrid control scheme to prevent the test fixtures from colliding. In the Tx test, Ry rotation was controlled at 0°, and in the Ty test, Rx rotation was controlled at 0°. Overall, this mechanical testing sequence (Figure 2) was designed to minimize disc volume loss (water exudation) during the protocol.^25^ The cycle amplitudes in each DOF test were chosen to be physiologically reasonable^30^, ^31^, ^32^: Tx = ±0.6 mm, Ty = ±0.6 mm, Rz = ±3°, Ry = ±4°, Rx = ±3°, and Tz = −0.2 MPa to −1.1 MPa.

### 2.3 Data analysis

For each DOF test, stiffness in the applied DOF (same‐axis stiffness) was calculated from the load‐displacement curve of the applied DOF in the final loading cycle (slow rate), as the first 4 cycles were considered preconditioning. Stiffnesses were calculated by linear regression between 70% and 90% of the maximum force or moment. Potential correlations between stiffness and each geometry parameter (height, lateral width, anterior‐posterior width, aspect ratio) were determined using Pearson correlation.

To determine the relationship (coupling) between each applied DOF and its off‐axis responses, the final loading cycle (a slow rate cycle) for the applied DOF was plotted against its corresponding off‐axis motions. To evaluate the strength of this correlation, a second‐order polynomial regression (*r* ^2^) was performed with significance set at *P* < 0.05. For the purposes of this study, data from the slow tests was used because the hexapod system controller exhibited poor control at the fast rate (future instrument improvements will address this).

**Figure 3:**
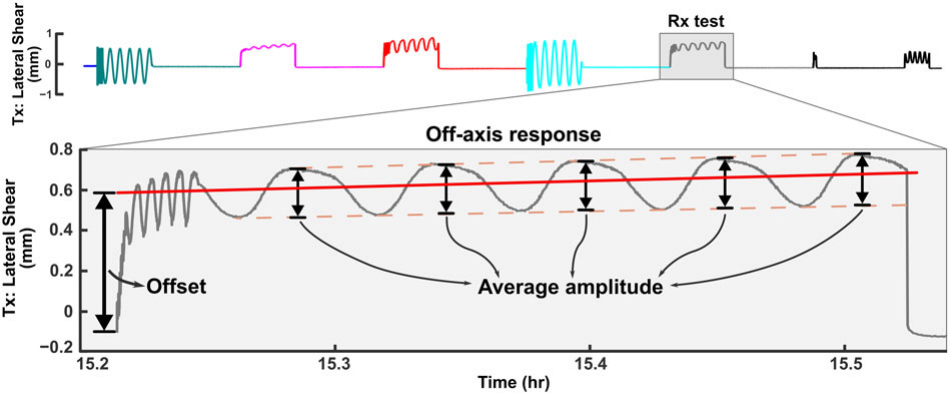
Representative response of Tx (lateral shear) throughout the entire test protocol. Large off‐axis responses to each DOF test can be observed. The expanded response of Tx to Rx loading (inset) shows how the offset and amplitude were calculated from the line that was fit through the oscillation (solid red line). The offset is the intercept of the line with the start of loading. The amplitude is the average distance from the line to the peak and valley of the cyclic response over the five applied cycles for each loading rate, calculated by subtracting the fit line from the experimental data

Since the applied loading and hence the off‐axis responses in each DOF test were cyclic, each off‐axis response was also characterized by the *offset* of its oscillations and the *amplitude* between its value at the start of the DOF test and the value about which it oscillated during the DOF test (Figure 3). The offset arises because the disc moves to a new reference position when a new cyclic loading pattern is applied. The amplitude and offset of each off‐axis response was determined relative to a line fit through the oscillitoryoff‐axis displacement/rotation vs time curve (Figure 3, red line). The offset is the intercept of this line with the start of loading. The amplitude is the average distance from the line to the peak and valley of the oscillation over the five applied cycles. To determine whether there was a significant off‐axis response, the offset and amplitude were compared to zero using a *t*‐test. To determine the effect of geometry, each geometry parameter was correlated against each off‐axis offset and amplitude.

## 3 RESULTS

### 3.1 Same‐axis mechanical response in the applied DOF

**Figure 4:**
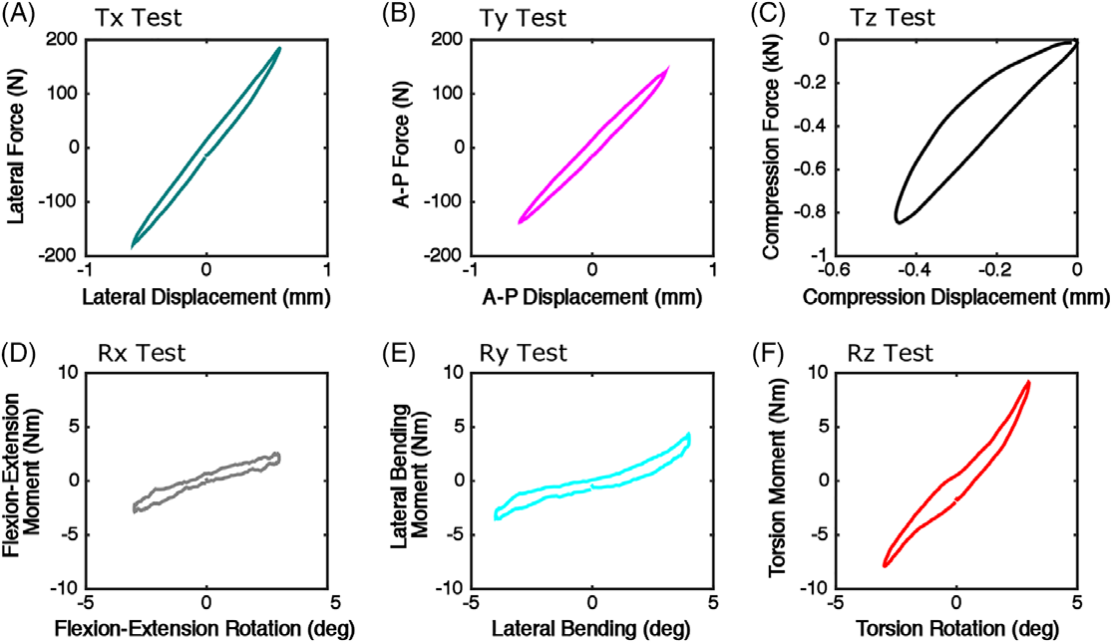
Representative same‐axis response of the final cycle for the slow rate in each DOF test. Top: the translation degrees of freedom (force vs displacement, A‐C), Bottom: the rotation degrees of freedom (moment vs rotation, D‐F)

**Figure 5:**
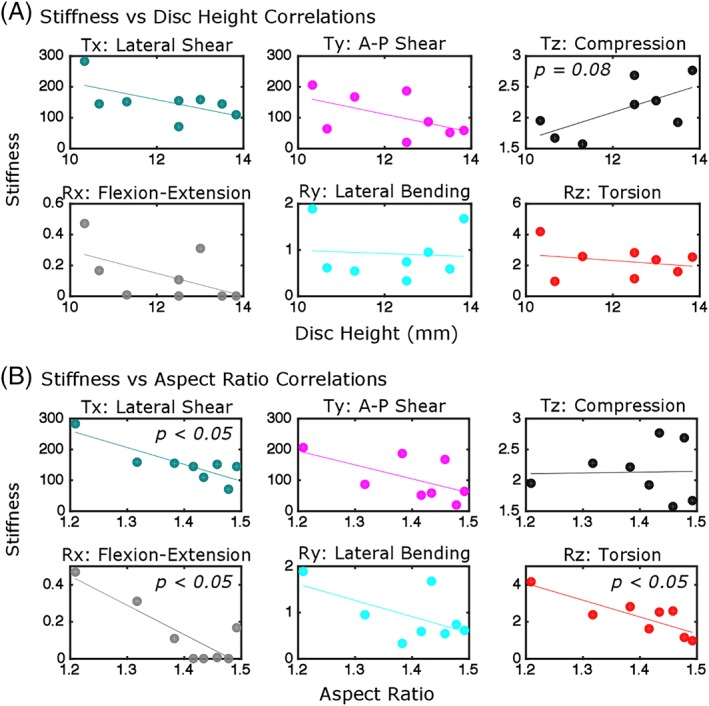
Correlations between stiffness and disc height (A) and aspect ratio (B). Stiffness for translation DOFs have units of N/mm (Tx, Ty) and kN/mm (Tz) while stiffness for rotation DOFs have units of Nm/deg. Correlations are listed in Table 1

A representative same‐axis load‐displacement (or moment‐rotation) curve for each DOF test's slow rate cycles is shown in Figure 4 and average same‐axis stiffnesses are reported in Table 1. Correlations of same‐axis stiffness with geometry were evaluated (Figure 5, Table 1). The axial compression (Tz) stiffness tended to correlate with disc height (*r* = 0.66, *P* = 0.08, Figure 5), and also strongly correlated with A‐P width (*r* = 0.75) and lateral width (*r* = 0.81), but not aspect ratio (*P* = 0.9, Figure 5B). Lateral shear (Tx) stiffness and A‐P shear (Ty) stiffness were not correlated with disc height (Figure 5). A‐P shear (Ty) stiffness correlated with lateral width (*r* = 0.70, *P* = 0.05), and lateral shear (Tx) stiffness (*r* = −0.85, *P* < 0.01) correlated with aspect ratio (Figure 5). Interestingly, none of the rotational DOF stiffnesses were correlated with disc height (Figure 5), but flexion‐extension (Rx) stiffness (*r* = −0.86, *P* < 0.01) and torsion (Rz) stiffness (*r* = −0.84, *P* < 0.01) were correlated with aspect ratio (Figure 5).

**Table 1:** Summary of results for slow rate tests

	Lateral Shear (Tx)	Anterior‐Posterior Shear (Ty)	Compression (Tz)	Flexion‐Extension (Rx)	Lateral Bending (Ry)	Torsion (Rz)
Overnight Creep	0.10 ± 0.12 mm	0.21 ± 0.26 mm	0.68 ± 0.34 mm	N/A	N/A	0.74 ± 0.61°
Stiffness	152.5 ± 60.8 N/mm	105.3 ± 70. 8 N/mm	2132.0 ± 437.7 N/mm	0.133 ± 0.176 Nm/deg	0.918 ± 0.568 Nm/deg	2.276 ± 1.033 Nm/deg
Correlation with Disc Height (*r* ^2^; *p*)	0.36; 0.12	0.28; 0.17	0.43; 0.08^#^	0.29; 0.17	0.01; 0.89	0.06; 0.55
Correlation with Aspect Ratio (*r* ^2^; *p*)	0.73; 0.01*	0.37; 0.11	0.00; 0.95	0.73; 0.01*	0.35; 0.13	0.70; 0.01*
Correlation with Lateral Width (*r* ^2^; *p*)	0.38; 0.10	0.50; 0.05*	0.66; 0.01*	0.19; 0.29	0.04; 0.63	0.09; 0.48
Correlation with A‐P Width (*r* ^2^; *p*)	0.01; 0.82	0.11; 0.43	0.57; 0.03*	0.01; 0.82	0.28; 0.18	0.04; 0.65

*P < 0.05, *#P* < 0.1.

### 3.2 Off‐axis mechanical response

**Figure 6:**
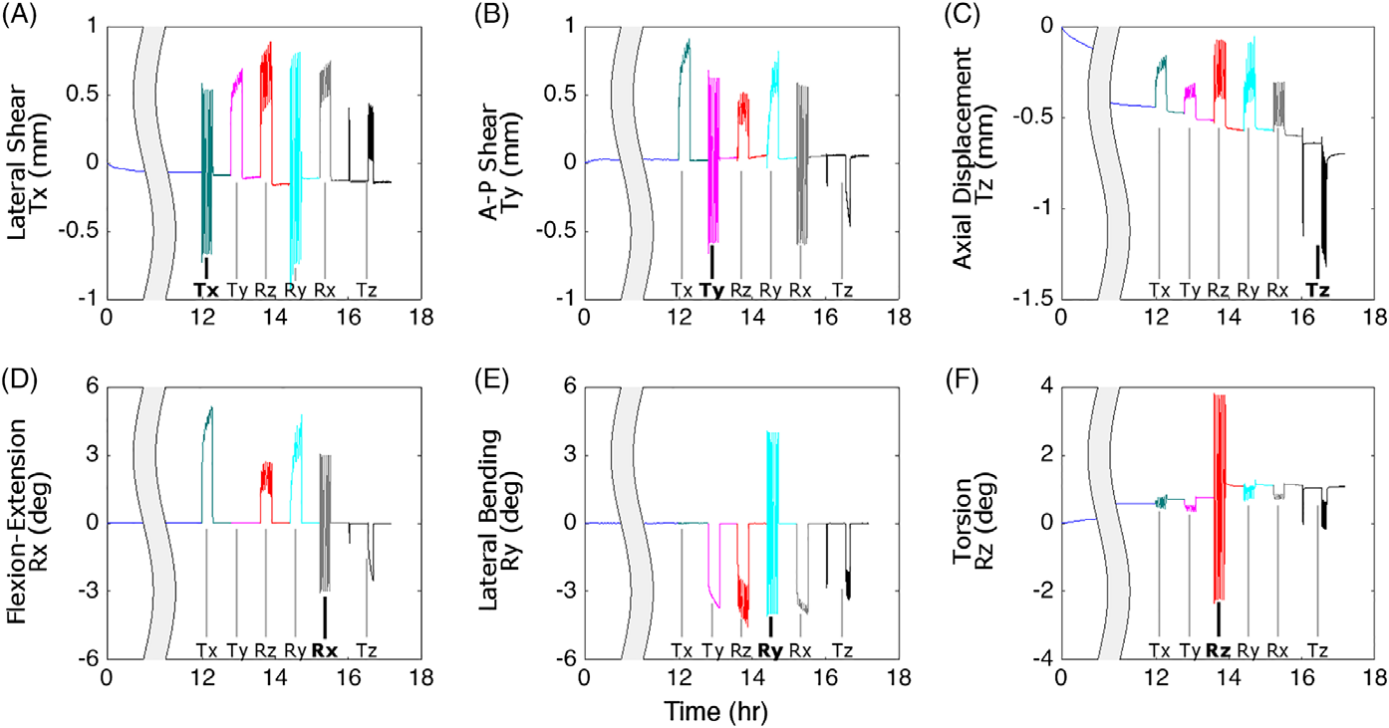
Representative displacements and rotations for all six DOFs throughout the entire test protocol. Labels on the bottom of each sub figure denote the DOF tests, with bold denoting the same‐axis response. Large creep occurred throughout the test for axial displacement (C) and some creep occurred for lateral shear (A), anterior‐posterior shear (B), and torsion (F), while flexion‐extension (D) and lateral bending (E) were controlled to zero throughout. Large off‐axis responses occurred for all six DOF throughout the duration of testing

The six DOF response for a representative specimen throughout the entire testing protocol is shown in Figure 6. During the 12‐hour preload (0.2 MPa axial compression), there was a large axial creep Tz = −0.70 mm (Table 1). A small amount of creep occurred in the lateral shear Tx = 0.10 mm, A‐P shear Ty = 0.21 mm, and torsion Rz = 0.74° axes (Table 1). Similarly, throughout the duration of testing the segment continued to creep in axial displacement (Tz, Figure 6C), with small amounts of additional creep in torsion (Rz, Figure 6F), lateral shear (Tx, Figure 6A) and A‐P shear (Ty, Figure 6B). There was no creep in flexion‐extension (Rx, Figure 6D) and lateral bending (Ry, Figure 6E), as these were controlled to be zero.

The off‐axis responses were large, with magnitudes similar to the applied DOF loadings (Figure 6). For example, lateral shear (Tx), was controlled to +/− 0.6 mm, but when the slow rate lateral bending (Ry) loading was applied, the coupled displacement in lateral shear ranged from +/−0.7 mm (Figure 6A). Similar effects, where the coupled off‐axis motion is greater than or equal to the applied motion, can be seen for all of the directions (Figure 6B‐E), except perhaps torsion Rz which is less than 1° (Figure 6F). Notably, the response in the axial direction was qualitatively different than that of the lateral and anterior‐posterior directions (Figure 6C). In the Tz DOF test, applied compressive loading caused the disc to decrease in height, but loading of the other DOFs caused disc height to increase in order to keep the axial force at its controlled preload value (Figure 6C). That is, when the disc was loaded in all other DOFs the disc pressure increases,^22^ and in order to maintain the applied preload, the axial compression decreases.

**Figure 7:**
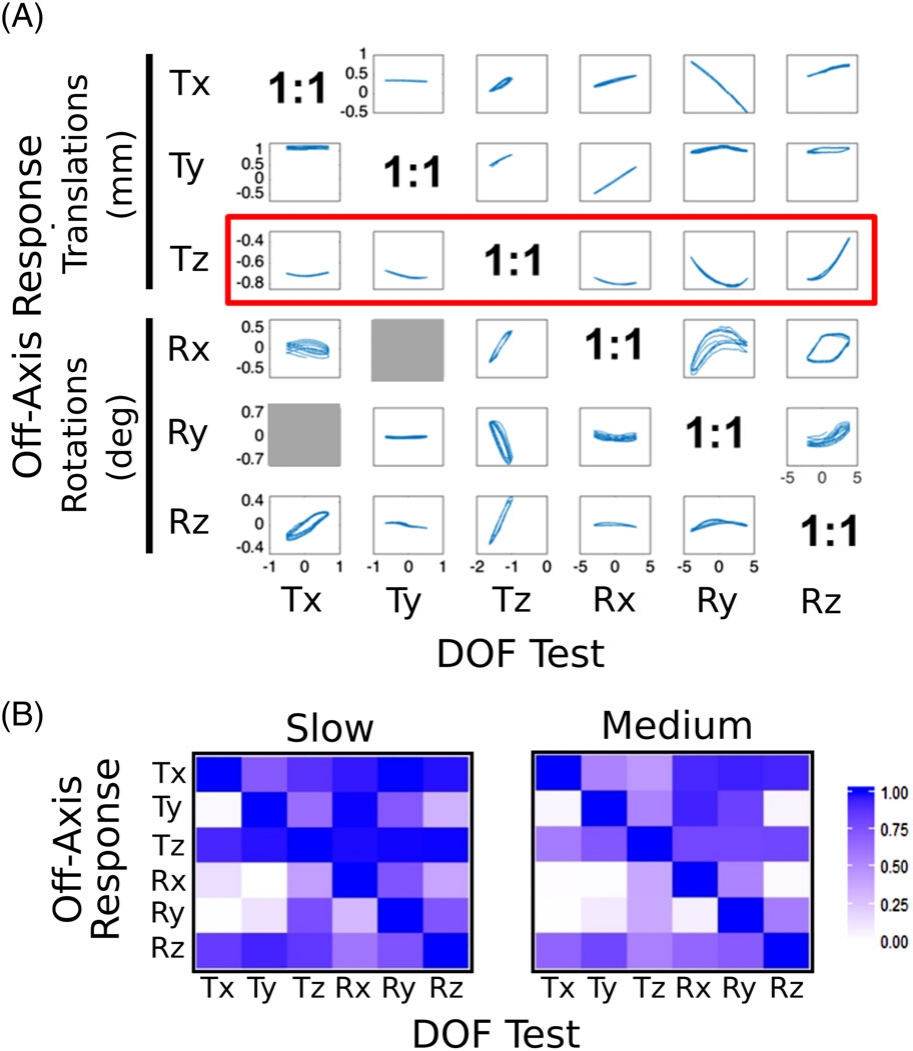
(A) Off‐axis response in each DOF averaged over all disc specimens plotted against the applied DOF for the slow rate. All responses were significantly correlated with applied loading (*P* < 0.01) except for Rx during Ty loading and Ry during Tx loading (which were controlled to be zero in the test protocol). (B) The strength of the correlations (*r* ^2^) between applied and off‐axis DOF for slow and medium rates of loading. The slow rate relationships are preserved at the medium rate although the *r* ^2^ values themselves are reduced

The average off‐axis responses were plotted against each applied DOF and tested for correlations (Figure 7). The off‐axis response was nonlinear and significantly correlated (*P* < 0.001) to applied displacements and rotations for all combinations and at both slow and medium rates (Figure 7B). Off‐axis rotations that were controlled to be 0° (Ry during the Tx test, and Rx during the Ty test) were not included; these are shaded gray in Figure 7A. The strength of the correlations ranged from *r* ^2^ ≈ 1 to ≈ 0 (Figure 7B). Off‐axis axial compression (Tz) was strongly correlated to all applied DOFs (red box in Figure 7A, *r* ^2^ > 0.8 in Figure 7B). The other off‐axis translations (Tx, Ty) tended to be especially strongly coupled to applied rotation (Rx, Ry, and Rz tests). For example, the off‐axis Tx response correlated with applied Ry and Rz loading with *r* ^2^ values of 0.99 and 0.98, respectively. In addition, we observed hysteresis in the off‐axis rotations that tended to be larger than in the off‐axis translations. The pattern of correlations between applied DOF loading and off‐axis responses was similar between the slow and medium loading rates, but with decreased *r* ^2^ values for the medium loading rate (Figure 7B).

### 3.3 Off‐axis amplitudes and offsets

**Figure 8:**
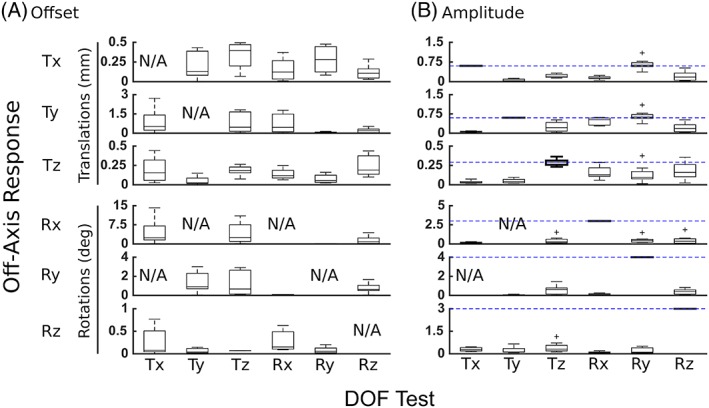
(A) Offset and (B) amplitude for the off‐axis DOFs, with median and inter‐quartile range for the slow rate shown. All offsets and amplitudes were significantly non‐zero. For comparison, the blue dashed line in (B) denotes the magnitude applied in that DOF's test. Amplitudes for translational DOFs tended to be larger when rotational DOFs are applied, and had magnitude similar to the applied amplitudes

The off‐axis responses in each DOF test was decomposed into an offset and amplitude (Figure 8). Many off‐axis offsets and amplitudes were large; all significantly differed from zero. A large Tz offset (0.19 mm) was observed in response to an applied axial torsion (Rz). Some off‐axis amplitudes were comparable to the applied amplitudes chosen for the DOF test protocols (Figure 8, blue dashed line). As in the off‐axis correlations (Figure 7), Tx and Ry, Ty and Rx, and Tz and Rz were found to be particularly strongly coupled. The off‐axis lateral shear (Tx) amplitude (0.62) was strongly coupled with applied lateral bending (Ry) and was similar to the amplitude applied in the Tx test (0.6 mm). Similarly, the off‐axis A–P shear (Ty) had a large amplitude (0.53 mm) and offset (0.46 mm) with applied flexion‐extension bending (Rx). In addition, the coupled off‐axis axial compression (Tz) amplitude (0.16 mm) with applied axial torsion (Rz) was large and of similar magnitude to the amplitude applied in the Tz DOF test (0.29 mm). The off‐axis Tz amplitudes overall tended to be greater in rotational DOF tests (0.10‐0.16 mm) than in translational DOF tests (0.03‐0.04 mm). These observations support the hypothesis that the disc's off‐axis displacements and rotations depend on (are coupled to) motion in the applied DOF.

### 3.4 Relationship between off‐axis mechanical response and disc geometry

**Figure 9:**
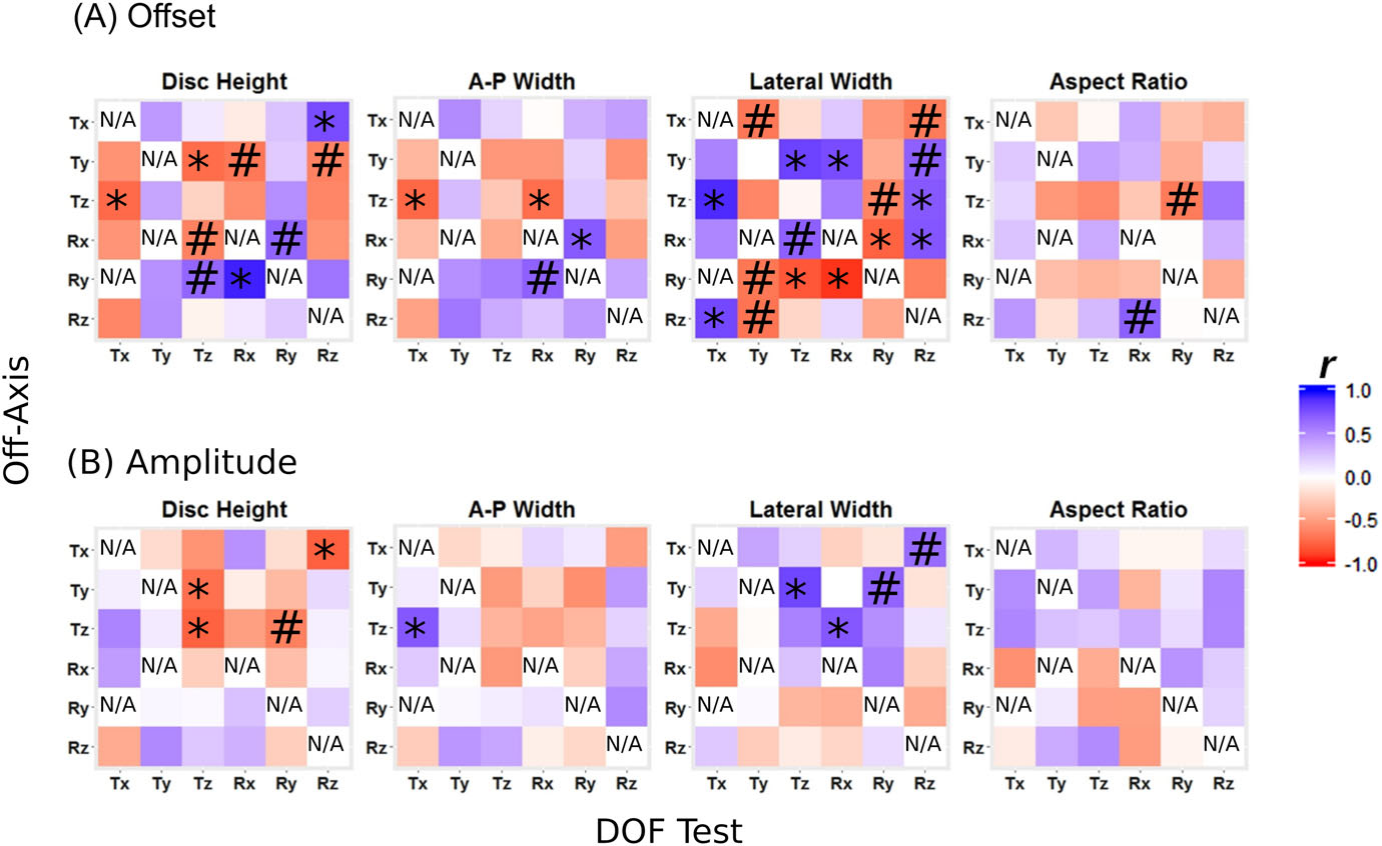
Heat maps showing correlations of disc geometry with offset (A) and amplitude (B) for each axis in each DOF test for the slow rate. Offsets were strongly correlated with geometry (top row), particularly lateral width, while amplitudes were generally not correlated with geometry (bottom row). * denotes *P* < 0.05, # denotes *P* < 0.1, and N/A denotes same‐axis loading and fixed loading (eg, Rx = 0 during the Ty test)

The off‐axis mechanical response in each DOF test was hypothesized to depend on disc geometry. Disc height, A‐P width, and lateral width were found to be significantly correlated with some, but not all, of the off‐axis offset and amplitude response variables (Figure 9). Aspect ratio was not significantly correlated with any off‐axis response. More offsets than amplitudes were significantly correlated with disc geometry. Lateral width had a particularly large number of significant correlations. Interestingly, lateral width and A‐P width tended to have opposed effects on the off‐axis offsets and amplitudes (opposite sign correlation coefficients).

## 4 DISCUSSION

This study quantified the mechanical behavior of the intervertebral disc in all six degrees of freedom, measured the coupling between applied loading and off‐axis motion, and evaluated the potential role of disc geometry in these mechanical behaviors. All off‐axis displacements and rotations were significantly correlated with the applied DOF and were of similar magnitude as physiologically relevant motion, confirming that off‐axis coupling is an important mechanical response. Interestingly, there were pairs of DOFs that were especially strongly coupled: lateral shear (Tx) and lateral bending (Ry), anterior‐posterior shear (Ty) and flexion‐extension (Rx), and compression (Tz) and torsion (Rz). The large offsets in lateral bending and flexion‐extension are consistent with the prior observation that bending and flexion generate the highest shear strains in the disc.^14^ Large off‐axis shears may contribute to injury risk in bending and flexion. Importantly, the large coupled motions observed in this study were caused by the disc alone, as the facet joints were removed. In addition, the disc responded to shear (Tx, Ty) and rotational loading (Rx, Ry, and Rz) by increasing in disc height in order to maintain the applied compressive load. This increase in disc height may be caused by an increase in intradiscal pressure during multiaxial loading.^22^

The average same‐axis stiffnesses in this study were comparable to those from those reported in literature for tests in lateral shear, A‐P shear, compression, and torsion.^8^ Flexion (0.13 Nm/deg) and bending (0.92 Nm/deg) stiffnesses tended to be lower than those reported in the literature for flexion (2.3 Nm/deg) and bending (3.4 Nm/deg) stiffness.^32^ This may be due to the lower compressive load (0.2 MPa) used in this study than in the other flexion and bending (0.4 MPa) tests. Axial load significantly affects the rotational and compressive stiffness of the disc.^33^ Studies which have used comparable axial follower loads (~0.2‐0.3 MPa) to those in this study have reported similar compressive stiffnesses^8^ while those which use larger follower loads have a corresponding higher stiffness.^32^ Axial loads during testing, and thus NP pressurization, are critical for disc mechanics. Comparisons between studies and application of reported values should be done with due attention to the axial loads applied and the expected resulting NP pressures.

This study demonstrated that disc geometry correlates with the same‐axis stiffness in all six DOFs and with some off‐axis responses. In particular, the disc's aspect ratio (lateral width/A‐P width) was a significant indicator of rotational and shear stiffnesses, whereas disc height was significantly correlated with compressive stiffness. In addition, lateral width was significantly correlated to each off‐axis offset. The correlation between lateral width and stiffness and off‐axis offsets may be related to the fact that lateral width is the largest dimension of the disc. It is the largest contributor to both the second area moment about z (resistance to torsion) and to the moment arm that resists lateral bending. The observed correlations demonstrate that geometry affects off‐axis DOF coupling and therefore contributes to the forces and moments generated in the disc during physiological loading. While not explicitly addressed here, the effect of spinal level and potential difference in geometry due to lordosis angles may also effect off‐axis responses. The relationship between geometry and mechanics is important for the validation and interpretation of finite element models. Geometry is a known indicator of disc mechanics^27^ and typically one geometry is used to represent the disc when developing a finite element model. When reviewing a model's predictions for multiaxial stiffnesses, one should put its simulated stiffness in the context of the model's geometry and the available data relating mechanics to disc geometry.

The discs used in this study were isolated discs without posterior elements, demonstrating that the isolated disc has large coupled motions even without the facets. Facets have a role in spine mechanics and may produce different off‐axis effects at the spine scale. Existing literature includes no information on the impact of facets on off‐axis responses, and there is conflicting reports regarding their impact on same‐axis stiffness. In position control tests, discs with facets had 60%‐100% higher compressive and rotational stiffness than those without,^33^ whereas in hybrid control tests, discs with facets showed no discernible difference in stiffness compared to isolated bone‐disc‐bone segments.^25^ Additional experiments with facets are needed to determine the relative role of the disc and the facets in six DOF mechanical responses. Another limitation of this study was the use of grade 2 to 3 mild/moderately degenerated discs. It remains unknown whether the coupled response measured here would be different with more advanced degeneration.

A strength of this study is the hybrid control approach used in the mechanical testing, where the applied DOF is position controlled (except for axial compression Tz) while the off‐axis is force‐ and moment controlled, with physiological axial compression applied throughout. Choices made when designing mechanical testing protocols, such as position vs load control, are known to effect the mechanical response.^25^, ^34^ Typical experiments control 1 to 2 DOFs. Some studies have investigated combined loading including compression and torsion,^22^, ^35^ combined rotational DOFs,^36^ and compression and flexion.^12^ One objective when choosing a testing protocol is to place it in the context of, and preferably simulate, in vivo conditions, thus enhancing translation of experimental results. Although in vivo loads are not completely known, it is known that the disc physiologically experiences six DOF loading from a combination of body weight and muscle forces.^37^, ^38^, ^39^ The six DOF hybrid testing system in this study has the key advantage of being able to replicate six DOF in vivo loading.^29^ Although this study perturbed each DOF in turn, these tests demonstrated that all six DOFs are coupled and that combined hybrid control and measurement of all six DOFs is essential to fully measure disc mechanics. A limitation of study is the relatively small sample size (*n* = 8) meaning that only the largest and most important effects were likely to be detected. There are several opportunities to further interrogate the experimental data obtained in this study and to interpret it with models. For example, future analyses could investigate the disc's six DOF viscoelastic mechanics, such phase lag, creep, hysteresis mechanism, and the effect of loading rate. Viscoelastic analysis may be useful to support future computational modeling work, which in turn would support simulation of between‐axis coupling in a wider variety of loading conditions.

The measured off‐axis coupling and their relationships with geometry are important to improve the design and evaluation of disc implants. Conventional design and evaluation of implants are limited to 1‐2 DOFs^40^ but this study showed that large off‐axis effects are generated during disc loading and that they are related to the disc's geometry. If not accounted for, these may contribute to implant failure, expulsion, or reduced function. Moreover, these results suggest the need for a patient‐specific approach to implant design in order to account for the correlation in mechanics with geometry. For example, bending in discs that have a larger lateral width may increase AF stresses and/or cause expulsion of the implant. Moreover, if an implant is designed without considering the off‐axis loads, the implant itself may fatigue due to large loads generated by the coupled off‐axes. Lastly, when a disc undergoes discectomy (changing the disc structure) or is replaced with an implant (changing the material), the stiffness in each of the six DOFs and their coupled off‐axis responses will be altered. These factors should be considered in the design and evaluation of disc implants and other treatments.

In conclusion, this study quantified strong mechanical coupling between DOFs in six DOF hybrid‐control mechanical tests of the intervertebral disc and demonstrated the mechanical coupling that varied with disc geometry. This multiaxial coupling and its interaction with geometry should be addressed in the design and evaluation of disc implants. This will likely require increased usage of multiaxial test equipment and consideration of patient‐specific disc geometry. Combined control and measurement of all six DOFs is needed to account for the multiaxial loads and their coupling experienced by the in vivo disc, and hybrid control multiaxial testers are well‐suited for this application.

## CONFLICTS OF INTEREST

The authors have no conflicts of interest to declare.

### Author contributions

All authors have read and revised the manuscript and have contributed to the study design and data analysis.
